# Guppies in large groups cooperate more frequently in an experimental test of the group size paradox

**DOI:** 10.1098/rspb.2023.0790

**Published:** 2023-07-12

**Authors:** Rebecca F. B. Padget, Tim W. Fawcett, Safi K. Darden

**Affiliations:** Centre for Research in Animal Behaviour, Department of Psychology, Faculty of Health and Life Sciences, University of Exeter, Exeter, UK

**Keywords:** volunteer's dilemma, group size, cooperation, anti-predator behaviour, Trinidadian guppies

## Abstract

The volunteer's dilemma, in which a single individual is required to produce a public good, predicts that individuals in larger groups will cooperate less frequently. Mechanistically, this could result from trade-offs between costs associated with volunteering and costs incurred if the public good is not produced (nobody volunteers). During predator inspection, one major contributor to the cost of volunteering is likely increased probability of predation; however, a predator also poses a risk to all individuals if nobody inspects. We tested the prediction that guppies in larger groups will inspect a predator less than those in smaller groups. We also predicted that individuals in larger groups would perceive less threat from the predator stimulus because of the protective benefits of larger groups (e.g. dilution). Contrary to prediction, we found that individuals in large groups inspected more frequently than those in smaller groups, but (as predicted) spent less time in refuges. There was evidence that individuals in intermediate-sized groups made fewest inspections and spent most time in refuges, suggesting that any link between group size, risk and cooperation is not driven by simple dilution. Extensions of theoretical models that capture these dynamics will likely be broadly applicable to risky cooperative behaviour.

## 1. Introduction

Animals can incur both costs and benefits from associating with others. Individuals in larger groups suffer greater competition and increased risk of disease compared to those in smaller groups (e.g. [1,2]), but there is evidence that individuals in larger groups have access to more information and resources (e.g. [3,4]), create greater confusion for predators [5,6] (though there is recent evidence to the contrary [7]), and are less likely to be attacked if there is a predation attempt due to selfish herd geometry and the dilution effect [8] (e.g. [9]). Group size can influence the interactions among individuals within a group, in both dynamic fission–fusion societies and stable groups. For example, mechanisms that can promote cooperative interactions, such as reciprocity and partner choice, can be more difficult to maintain when there are more potential interacting partners [10]. Cooperation among individuals within groups often involves individuals collectively acting to allow exploitation of a resource or provide defence against predators. Cooperative hunting of large prey (e.g. in wolves and lions [11]) and cooperative inspection of potential predators (e.g. in small fish and some ungulates [12,13]) are both examples of these scenarios. These are often termed ‘public goods' scenarios (or public goods ‘games’) because the benefit from the behaviour is available to all individuals in the group, not just those who contributed to its production. While these behaviours provide a reward to all individuals in the group (e.g. food and information, as in the above examples), they are costly (risky) to perform. An individual would therefore appear to gain the most if it did not participate while others did. However, for an individual who does not participate, there is no guarantee that the reward is produced.

Some of these public goods scenarios can be modelled as a ‘volunteer's dilemma’. The volunteer's dilemma is similar to an N-person Prisoner's Dilemma, except that a threshold number of individuals (usually modelled as one) is required to produce the total public good for the group. These models are occasionally referred to as N-person Prisoner's Dilemmas in some parts of the literature (e.g. [14]) but here, we consider them a distinct framework because models produce qualitatively different predictions when the benefit function is a nonlinear function of the number of cooperators (e.g. a step function, as in the volunteer's dilemma) compared to a linear function (as in the N-person Prisoner's Dilemma [15]). When the benefit is a step function (or other nonlinear function) of the number of cooperators, it can pay to cooperate even when nobody else does [15–17]. The evolutionarily stable state is therefore a mixture of cooperation and defection, with the proportions of each depending on the relative values of the costs of volunteering or not volunteering [15,16]. Because an individual's best strategy if nobody else cooperates is to cooperate, the dilemma is centred around a trade-off to minimize both the cost incurred by volunteering and the cost incurred if nobody volunteers (meaning the public good is not produced [18,19]). Paradoxically, the volunteer's dilemma (along with several other public goods models, discussed in [20,21]) predicts that as group size increases, there is a lower probability that the public good is produced because there is a reduced probability that any one individual volunteers [22]. Smaller groups might therefore be more successful than larger groups at producing public goods in a volunteer's dilemma scenario.

Empirical results examining the effect of group size on cooperative behaviour are largely limited to human studies, and can depend on precise details of the experiment. For example, Nosenzo *et al*. [23] found that high-reward games resulted in individuals in large groups cooperating less than those in small groups (as predicted), but this was reversed when the reward was low. Similarly, Barcelo & Capraro [24] found that when individuals had the choice to invest different amounts in a common good, individuals in larger groups were more cooperative, but when individuals only had the choice to invest all or nothing in the common good, individuals in smaller groups cooperated more. Empirical tests of the effect of group size on cooperation in animals are primarily carried out in the context of vigilance in groups, and vigilance is usually found to decrease with group size, as predicted by the volunteer's dilemma (e.g. [25,26]). Predator inspection shares several features in common with vigilance in groups, because both involve individuals paying a personal cost and producing a collective benefit. We would therefore predict that the effect of group size on both types of behaviour might be similar, but the effect of group size on predator inspection has not been tested.

In this experiment, we used predator inspection as an experimental paradigm to test the prediction from the volunteer's dilemma that individuals in larger groups would be less likely to cooperate than those in smaller groups. During predator inspection, one or more individuals breaks away from the relative safety of the group to inspect a potential predator [12,13]. This behaviour is thought to be risky because individuals reduce the distance between themselves and a potentially dangerous predator, increasing the probability that they are attacked [13,27,28]. However, predator inspection also confers benefits, providing information [13] that might help with escape, and potentially deterring predators [29]. It can also speed up the return to foraging [30], if individuals can determine that there is no threat. Because the information and deterrent effect from predator inspection appears to be available to the entire group, not just the inspectors, we consider inspecting to be a cooperative behaviour [31,32]. Information gained during predator inspection can be transferred from a single individual to many others and the benefit from predator inspection has therefore previously been modelled as a step function of the number of cooperators, with a single individual needed to produce the public good [14]. As such, we propose that the decision to inspect a predator in a social context fits within the conceptual framework of the volunteer's dilemma.

Our experiment was carried out using Trinidadian guppies (*Poecilia reticulata*; hereafter ‘guppies’), a species of tropical freshwater fish, as a model system. Predation is considered to be a major driver of morphology and behaviour in guppies (e.g. [33–35]). In particular, predation on guppies by piscivorous fish is thought to have driven the evolution of predator inspection, which is observed in all populations of guppies [36], and appears to play a role in the social structure of wild populations [37,38]. Guppies live in highly dynamic, fission–fusion societies and occupy a wide range of naturally occurring shoaling group and pool population sizes [37], making them an ideal subject for the study of group size and cooperation.

In this laboratory experiment, we measured the frequency, duration, and distance of inspections as well as the overall proportion time spent inspecting for individual female guppies in three different experimental group sizes, operationalized as pool-level population size. We also measured group-level cohesion, and individual refuge use as measures of risk perception and performed an exploratory analysis on sub-group formation to identify whether shoaling sub-group sizes differed across experimentally manipulated group sizes. We predicted that individuals in larger groups would inspect less frequently and would spend less time in refuges.

## 2. Methods

This study was pre-registered prior to the collection of data. The pre-registration can be found at: https://doi.org/10.17605/OSF.IO/UR9H6 [39]. Our methods followed our pre-registered plan, except for three additional exploratory analyses: mean distance during inspection, sub-group size, and number of inspectors per inspection. Data are available at: https://doi.org/10.5061/dryad.2fqz612v3 [40]. Code is available at: https://github.com/beckypadget/groupsize_analysis.

### (a) Guppy populations

Guppies used in the experiment were laboratory-bred guppies reared at Washington Singer Laboratory, University of Exeter, UK. The guppies used were descended from guppies originally collected in 2013 from a high-predation region of the Guanapo river on the island of Trinidad. Guppies for all but one trial were reared from fry in a single large (65 l) stock tank containing approximately 250 fish, both males and females of mixed ages. Males and females were allowed to interact. This setup was used to facilitate natural social encounters and allow the guppies to gain some familiarity with their experimental group-mates to more closely mimic natural conditions. For logistical reasons, in one trial, guppies were collected as adults from smaller (17 l) stock tanks containing males and females of mixed ages; two guppies were collected per tank. Trial conditions were otherwise identical. All housing tanks contained a small amount of gravel substrate and plastic plant refuges. Guppies were fed with flake food twice per day between 2 and 3 h prior to the trial, and immediately after.

### (b) Experimental design

Trials were conducted once per day over a 17-day period in November and December 2021 at the Washington Singer Laboratory, University of Exeter, UK. Guppies in experimental group sizes of 5, 10, and 20 individuals were allowed to interact with a model predator stimulus for 8 min, following a 30-minute acclimatization period, in a 1.5 m × 1.5 m pool filled to a height of roughly 10 cm with fresh water (between 23.6°C and 25°C; no more than 0.5°C different from that of their home tank). Trials were conducted under full-spectrum light of approximately 35 lux, diffused by hanging white PEVA shower curtains from an overhead rail approximately 1.75 m above the pool. Trials were videoed using a Sony FDR-AX53 4K video camera mounted on the overhead rail above the centre of the trial pool.

#### (i) Predator stimulus

We used a model of a predator instead of a live predator fish in order to standardize the stimulus across trials. The predator model was made using polymer clay (Fimo granite effect; cured in a domestic oven at 110°C for approximately two hours). It was approximately 17 cm long and 5.5 cm in diameter, similar in shape to a pike cichlid (*Crenicichla alta*), an important predator of adult guppies in the wild. Characteristic pike cichlid stripes (of black coloration to the human eye) and eyes (of orange coloration to the human eye) were added using chalk pastel applied after curing. The model was soaked in fresh water for 48 h prior to the experiment to ensure that no particles could enter into the trial pool water. The predator model was suspended in the water using 1.5 mm transparent fishing line tied to a small hole in the dorsal fin. The tail was attached—also via fishing line—to a servo, controlled by an Arduino UNO-style board (electronic supplementary material, figure S1). This allowed the predator model to move slowly forwards and backwards, emulating the movement of a live pike cichlid maintaining its spatial position (wiring diagram in electronic supplementary material, S2).

To allow the predator model to be revealed and concealed when needed, a Perspex screen was placed around the model. The screen was triangular and consisted of three sheets of opaque, white Perspex (measuring 400 × 120 × 3 mm (×2) and 200 × 120 × 3 mm), fixed at the corners with marine-grade silicone and covered with gravel-patterned vinyl; the bottom and sides of the trial pool were also covered with the same gravel-patterned vinyl. The screen was suspended in the water using fishing line linked to a pulley and motor (controlled by the Arduino UNO-style board), which was used to raise the screen and reveal the predator. This mechanism allowed the screen to be raised at a consistent speed without the need for a researcher to move around the pool, potentially causing disturbance.

#### (ii) Protocol

For each trial, fish were removed from their holding tank in groups. They were transferred in water to the trial pool, where they were allowed to acclimatize: the transfer tank (2.5 l) was initially placed into the pool and guppies were kept in the (covered) tank in the pool for 15 min before being released into the pool and allowed to settle for a further 30 min. The barrier around the predator model was then raised (by remote activation of the motor, except in two trials in which the mechanism failed and the screen was raised manually) such that it did not break the water surface, and the predator model was visible to the guppies. The predator model movement (controlled by the servo) was activated simultaneously (also remotely). The screen remained raised for 8 min. After 8 min, the screen was lowered (provided that no fish were within approximately 30 cm of the screen).

### (c) Data collection

Spatial position data for individuals were collected using TRex automated tracking software (after pre-processing using the related TGrabs software [41]). TRex uses kinematics and individual recognition (via deep learning) to track and identify individuals from videos. The primary output of the software is a dataset of *x*, *y* coordinates for each recognized individual in each frame of a video (videos were recorded at 25 frames per second). We used mostly default parameters (as is recommended); the parameter values that we changed to track the fish more accurately are given in electronic supplementary material, table S1.

We also used TRex to identify the positions of the predator model in each video. We did this for each video to get accurate positions of the predator model in each trial because there were small differences in camera and apparatus position between trials, which would have compromised the accuracy of our measures if we had assumed a fixed model location across the videos.

The tracking data outputted from TRex were processed to exclude impossible tracks—those for which the predicted speed was above 50 cm s^−1^, or the next point was more than 50 cm (Euclidean distance) from the previous point. Missing frames, where the software failed to detect any individuals in a frame, were excluded.

Inspection behaviour data were collected from 7 min of video that began 30 s after the screen first began to lift (determined manually for each video). A delay of 30 s was chosen to reduce noise caused by variation in screen movement during lifting; the screen would be fully lifted and most screen movement stopped after 30 s. For each fish, we collected the following data on inspection behaviour: number of inspections; duration of inspections; overall proportion of time spent inspecting; mean distance of each inspection; and the distance (to any part of the predator model) of an individual's closest inspection (electronic supplementary material, table S2). To investigate risk perception outside of inspection events, we measured the time that individuals spent in refuges. Because fish were not visible when in the refuge, we used the proportion of time that the individual was not visible (out of the full data collection period) as a measure of the time in the refuge. To investigate cohesion, we collected group density (median of 1/Voronoi area for each fish [42]) from the same 7-minute data collection period as the inspection data (the ‘during’ phase, while the predator model was visible), and also from the 7-minute period immediately prior to the screen beginning to lift (the ‘before’ phase).

### (d) Statistical analysis

#### (i) Inspection behaviour

We used CmdStanR to fit Bayesian generalized linear mixed models (GLMMs) using Markov chain Monte Carlo [43], and fit models to each behaviour separately. Experimental group size (a factor with three levels) was included as a ‘fixed’ effect in all models. Water temperature and the mean length of the fish in the experimental grouping (both continuous) were also included as ‘fixed’ effects in all models to account for differences in overall activity caused by slight differences in water temperature and mean fish size between trials. Trial ID (a factor with 15 levels unique to each trial, reflecting the nested design) was included as a random effect term in the models to account for the non-independence of fish in the same experimental group.

We modelled count data (number of inspections) as coming from a Poisson distribution and proportion data (proportion time inspecting) as a Beta distribution (following [44]). For duration of inspections and minimum inspection distance, we used a Gamma regression. For mean distance of inspections, we used a Beta regression because the distribution was bounded at 30 cm (outside of which we did not consider a fish to be inspecting). We used weakly informative priors for all parameters: for the Poisson model: *b* ∼N(0*,*1); *σ* ∼N(0*,*1); for the Beta models: *b, σ* ∼N(0*,*3) and *κ* ∼N(0*,*5)); for the Gamma models: *α,* intercept*, β*_group size_*, β*_temperature_ ∼N(0*,*1)). We visually checked the fit of all model predictions to the original data.

To quantify the evidence for the effect of experimental group size on the metrics that we measured, we calculated the Savage–Dickey Bayes factors. The Savage–Dickey Bayes factor gives a measure of the evidence for the model given the data. This is found by calculating the ratio between the prior density and the posterior density, which (counterintuitively) gives the probability ratio in favour of the posterior density such that a higher value indicates stronger evidence for an effect of the parameter [45]. We interpreted Bayes factors roughly in line with Jeffreys [46] and Kass & Raftery [47]: a Bayes factor between 1 and 3 is weak evidence; between 3 and 10 is moderate evidence; between 10 and 30 is strong evidence; between 30 and 100 is very strong evidence; and *greater than* 100 is extreme evidence in the direction stated—for or against an effect of the parameter.

For clarity of interpretation, for categorical variables we report the between-group contrasts as the median of the back-transformed difference in posterior distributions, and we report the back-transformed 89% highest density interval (HDI) to show the likely effect size and range, respectively, of each parameter in its original scale. For pool temperature and fish length (continuous variables) we report effect size estimates and 89% HDI, as the back-transformed median and 89% HDI of the posterior samples in electronic supplementary material, table S3.

#### (ii) Risk perception

*Refuge use.* Because the tracking software could not track individuals when they were under plant cover or over the gravel, we used visibility (to the software) of each individual as a measure of refuge use for that individual. We recorded the proportion of the 7-minute data collection period that each individual was visible in both the ‘before’ phase (predator stimulus not yet visible) and ‘during’ phase. We then calculated 1 minus the proportion of time visible to give the proportion of time that the individual was *not* visible, i.e. was in a refuge. We modelled these data as coming from a Beta distribution, using weakly informative priors: *b,σ* ∼N(0*,*1) and *κ* ∼N(0*,*20)). We included the interactions between experimental group size and phase as fixed effects in the model to determine whether individuals in different experimental group sizes differed in their refuge use, and whether this depended on phase. We also included pool temperature and mean fish length as fixed effects to account any differences in these factors among trials. Trial ID was included as a random effect.

*Cohesion.* To measure cohesion, we used the tracking data to calculate the area of the Voronoi polygon around each fish for each second (using the deldir package in R [48,49]), before calculating each individual's ‘density’, 1*/*Voronoi area, each second. We then used the median of the values for each fish to calculate a group-level measure of density each second, before aggregating this (using the median) over each minute to create a 14-minute time series (7 min in the ‘before’ phase when the predator stimulus was not visible, and 7 min in the ‘during’ phase when it was visible). We aggregated data because the fish were not visible to the tracking software when they were in refuges.

In an exploratory analysis, we investigated whether individuals in different-sized experimental groups (5-, 10- and 20-fish manipulations) formed sub-groups of different sizes before and after introduction of the predator stimulus. The aim of this analysis was to determine the actual shoaling sizes within each experimental group size and whether this differed across experimental group sizes. We used the FPC package in R [50] to calculate the number of ‘clusters’ (sub-groups) that the fish had formed using DBSCAN with a reachability distance of 6 cm (approximately four guppy body lengths [51]). This is approximately equivalent to the ‘chain rule’ [52], but does not consider lone individuals to be a cluster. We then calculated the number of fish in each of the clusters and calculated median cluster size per second, then aggregated this (using the median) for each minute. This gave us a time series of median sub-group size over 14 min (7 in the ‘before’ phase, and 7 in the ‘during’ phase).

Because neither density nor sub-group size are comparable among groups of different sizes (larger groups in the same space will on average be more dense, and form larger sub-groups), we tested whether individuals in any of the experimental group sizes changed their density or sub-group size after the predator stimulus was revealed, compared to before it was revealed. For both density and sub-group size, we fitted separate GLMMs (in CmdStanR) to identify whether any groups changed their behaviour after the predator stimulus was revealed. In both models, we included a smooth term describing a Gaussian process, which accounted for temporal autocorrelation arising from measuring the same animals over time. We modelled group density data as coming from a Beta distribution, and sub-group size (counts of individuals in a sub-group) as a Poisson distribution. We included experimental group size, pool temperature, and mean fish length as ‘fixed’ effects. We additionally included a term for phase and its interactions with experimental group size. These interactions allowed us to identify whether groups of different sizes responded differently to the predator stimulus. We also included, as a random effect, the trial ID. As above, we report the Bayes factors, the 89% HDI and point estimate of the marginal effect on the scale of the data for experimental group size.

#### (iii) Number of inspectors per inspection for each experimental group size

Due to simple numerical effects, we would expect to see more inspectors per inspection in larger groups. We ran a simple Poisson family generalized linear model with group size as the only predictor to identify (non-causally) how number of inspectors changed with experimental group size.

## 3. Results

### (a) The effect of experimental group size on inspection frequency and behaviour

**Figure 1.**
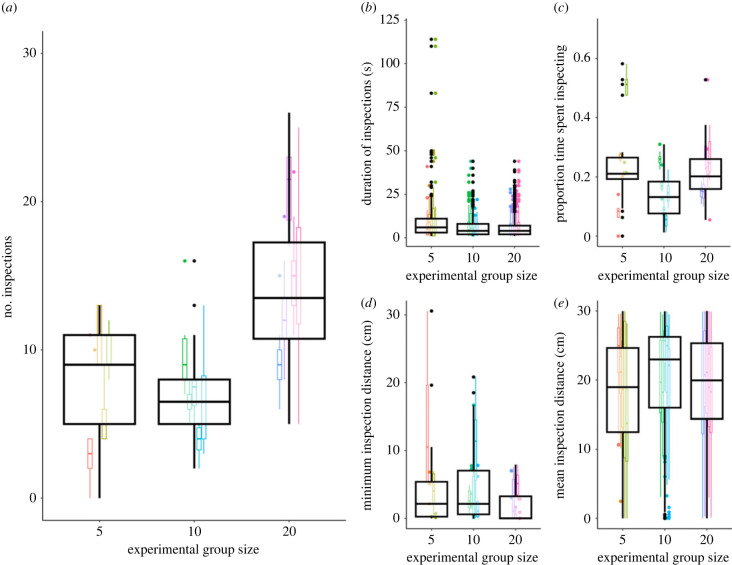
Individual inspection frequency and behaviour. The effect of experimental group size on an individual's: (*a*) number of inspections; (*b*) duration of inspections; (*c*) proportion of time spent inspecting; (*d*) minimum inspection distance; and (*e*) mean inspection distance. Large boxes show data for all trials of that experimental group size; small boxes show each trial (colours correspond to trials). We found that individuals in large groups inspected more frequently, but that no other measured aspect of inspection behaviour was affected by experimental group size.

Individuals in large groups inspected more frequently than those in both intermediate groups (moderate evidence) and small groups (weak evidence; figure 1; table 1). Individuals in intermediate and small groups inspected roughly the same number of times. There was moderate evidence *against* an effect of experimental group size on inspection duration. There was also no effect of experimental group size on the proportion of time spent inspecting, and no evidence that experimental group size impacted the minimum or mean distance of inspections. Direct comparisons among experimental group sizes are given in table 1. Neither pool temperature nor mean fish length were related to any of the behaviours (electronic supplementary material, table S3).

**Table 1.** Effect of experimental group size on individual inspection behaviour. Bayes factor, 89% highest density interval (HDI) and marginal effect for each experimental group size comparison for each inspection behaviour. The Bayes factor indicates the strength of evidence and the ‘direction’ of the Bayes factor denotes whether this evidence is in favour of an effect or against an effect of experimental group size. The 89% HDI and marginal effect are calculated from the back-transformed posterior distribution and show the range of likely effect sizes and median effect (respectively) of moving to the larger group; both are given on the scale of the data in the stated units. Results in bold indicate moderate (or higher) evidence in favour of an effect of experimental group size. Results in italics show where the 89% HDI does not overlap zero, suggesting a potential small effect in either a positive or negative direction.

behaviour	groups compared	Bayes factor, direction (strength)	89% HDI	marginal effect
number inspections	small → intermediate	5.0, against (moderate)	−3.41, 2.12	−0.56 inspections
	*small* → *large*	*1.7, in favour (weak)*	*1.03, 10.83*	*6.2 inspections*
	**intermediate → large**	**9.5, in favour (moderate)**	**2.55, 10.98**	**6.8 inspections**
duration inspections	small → intermediate	5.6, against (moderate)	−2.18, 0.34	−0.89 s
	small → large	8.5, against (moderate)	−1.81, 1.32	−0.27 s
	intermediate → large	7.6, against (moderate)	−0.56, 1.79	0.61 s
proportion time inspecting	small → intermediate	2.2, against (weak)	−21, 18	−0.80%
	small → large	1.9, against (weak)	−19, 25	2.80%
	intermediate → large	2.0, against (weak)	−18, 24	3%
minimum distance	small → intermediate	1.3, against (weak)	−1.79, 3.82	0.84 cm
	small → large	1.3, against (weak)	−2.47, 1.45	−0.29 cm
	intermediate → large	1.1, in favour (weak)	−4.23, 1.17	−1.1 cm
mean distance	small → intermediate	4.5, against (moderate)	−2.4, 3.9	−0.06 cm
	small → large	3.7, against (moderate)	−5.1, 2.7	0.09 cm
	intermediate → large	3.3, against (moderate)	−5.1, 1.2	1.8 cm

### (b) The effect of experimental group size on risk perception

#### (i) Refuge use

**Figure 2.**
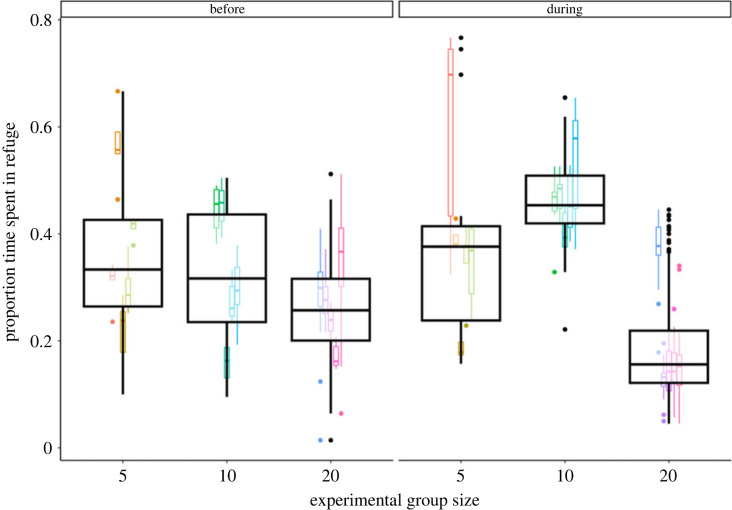
Individual refuge use. The proportion of time that individuals spent in refuges in different experimental group sizes ‘before’ (left) and ‘during’ (right) the appearance of the predator stimulus. Large boxes show data for all trials of that experimental group size; small boxes show data for each trial; colours represent different trials. Individuals in larger groups spent less time in refuges while the stimulus was visible than individuals in small or intermediate-sized groups, and intermediate-size groups spent the most time in refuges.

While the predator stimulus was visible, there was evidence that individuals in large groups spent the least time in the refuges, while those in intermediate groups spent the most amount of time in refuges (figure 2; table 2). This effect of experimental group size was not present in the ‘before’ phase, when refuge use was unrelated to experimental group size (figure 2 and table 2).

**Table 2.** Effect of experimental group size and phase on time in refuge. Bayes factors, 89% highest density interval (HDI) and marginal effect for each experimental group size. The Bayes factor indicates the strength of evidence and the ‘direction’ of the Bayes factor denotes whether this evidence is in favour of an effect or against an effect of experimental group size. The 89% HDI and marginal effect are calculated from the back-transformed posterior distribution and show the range of likely effect sizes and median effect (respectively) of moving to the larger group; both are given on the scale of the data as a percentage of time. Results in bold indicate moderate (or higher) evidence of an effect. Results in italics show where the 89% HDI does not overlap zero, suggesting a potential small effect in either a positive or negative direction.

contrast	Bayes factor, direction (strength)	89% HDI	marginal effect
small before → intermediate before	4.2, against (moderate)	−13, 8	−2.5%
small before → large before	3.5, against (moderate)	−16, 7	−4.2%
intermediate before → large before	4.4, against (moderate)	−12, 8	−1.7%
small during → intermediate during	1.7, in favour (weak)	−1, 21	10%
*small during* → *large during*	*1.7, in favour (weak)*	−*24,* −*2*	−*13%*
**intermediate during → large during**	**68, in favour (strong)**	−**33,** −**12**	−**23%**

#### (ii) Cohesion

There was no evidence that experimental groups of any size became more or less cohesive after the introduction of the predator stimulus (electronic supplementary material, table S4). Electronic supplementary material, figure S3, illustrates the Voronoi polygons at roughly equally spaced time intervals throughout the 14-minute data collection period.

#### (iii) The effect of experimental group size on sub-group size

We found that while there was wide variation in the sub-group size within trials, mean sub-group sizes were generally the same regardless of the experimental group size. Direct comparisons among experimental group sizes are given in electronic supplementary material, table S5.

### (c) Number of inspectors per inspection for each experimental group size

**Figure 3.**
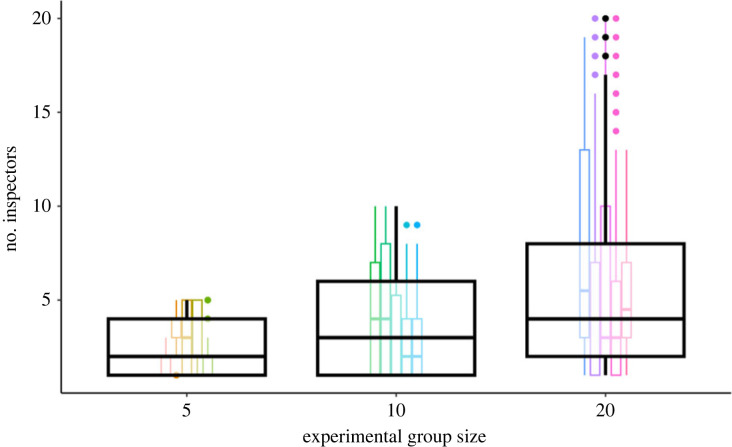
Number of inspectors per inspection for each experimental group size. The large boxes show aggregated data for each group size. The small boxes show data for each trial; colours correspond to trials. Number of inspectors in each inspection increases with group size.

We confirmed that there were more inspectors in the larger groups (5 fish estimate: 0.93, *p* = 2 × 10^−16^; 10 fish estimate: 1.3, *p* = 2 × 10^−16^; 20 fish estimate: 1.7, *p* = 2 × 10^−16^; figure 3).

## 4. Discussion

In this experiment, we tested the prediction of the volunteer's dilemma that individuals in larger groups would be less likely to cooperate (inspect a predator stimulus) than those in smaller groups. We found that individuals in large (20-fish) groups were not less cooperative than those in smaller (5-fish or 10-fish) groups—they actually cooperated more frequently than individuals in smaller groups. Before our experiment, we speculated that individuals in larger groups might cooperate less because they perceived the risk posed by the predator stimulus to be lower, and individuals would thus perceive a cost–benefit trade-off less favourable for cooperation than individuals in smaller groups where there would be a greater cost of no one cooperating. Individuals in large groups did appear to perceive a lower level of risk (based on time spent in refuges), but nevertheless cooperated more frequently than those in smaller groups. This would suggest that increased (perceived) risk limits rather than promotes participation in this anti-predator behaviour. Alternatively, it might indicate that larger groups are able to use the shoal itself as a refuge, remaining outside of environmental refuges while perceiving a similar (or lower) level of risk. There was weak evidence that the effect of group size on number of inspections and risk perception was non-monotonic with individuals in intermediate-sized groups performing the fewest inspections and having the highest levels of refuge use. Guppies did not adjust cohesion or sub-group size in response to experimental group size or presence of the predator stimulus, suggesting that these measures of shoal cohesion might not be informative measures of risk perception in our experiment. We also found no effect of experimental group size on the duration of inspection, overall time spent inspecting, or inspection distance (either minimum or mean). These results suggest that guppies do not behave as predicted by the volunteer's dilemma in relation to group size in the context of predator inspection. Perception of the risk posed by a predator appeared to correlate with the decision to inspect, but not behaviour during inspection, further suggesting that perception of risk before inspecting is not related to behaviour during an inspection. The potential non-monotonicity of the effect of group size indicates that the effect of group size seen in this experiment might not be reflective of a simple dilution effect.

The increased individual frequency of cooperation that we saw in large groups in this experiment suggests that guppies can adjust their investment in cooperative anti-predator behaviour and might do so partly in response to the number of other individuals nearby. We also found that our measure of risk perception was affected by group size, with individuals in large groups showing lower levels of refuge use, suggesting that they perceived the risk posed by the predator stimulus to be lower. This reflects empirical literature that suggests individuals in larger groups are at lower risk of being eaten by a predator [53]. Because it reduces the risk posed by the predator, being in a larger group is likely to reduce the cost to an individual if nobody inspects because if the predator attacks successfully, each individual has a reduced chance of being the attacked individual (e.g. because of dilution and herd geometry [8,53]). However, this would predict that individuals in large groups would inspect less (as in the volunteer's dilemma), not more.

More frequent inspections in larger groups could suggest that the cost of cooperation is also reduced in larger groups. We found that individuals in all experimental group sizes inspected at the same distance from the predator, but in large groups, inspecting parties were larger. This could mean that each inspection was less costly to individuals in larger groups, because of the protective effects of group size acting during inspections [8,53]. This could help to explain why individuals in large groups inspected more frequently (if they were able to gain information on inspection party size prior to approaching the predator) but does not explain why behaviour during inspection was similar across group sizes, nor account for the potential non-monotonicity in the effect of group size. Models allowing the cost of cooperation to depend on group size do predict that individuals in larger groups will inspect more (see example in electronic supplementary material, S6.1), but this is unlikely to be a realistic condition in natural scenarios; instead costs are likely to be shared among cooperators only. However, theoretical models allowing the cost of volunteering to be shared among volunteers (e.g. [54]) do not reverse the negative effect of group size on volunteering. We also found weak evidence that the effect of group size on the number of inspections was non-monotonic, but we would expect this effect to be monotonic if it was driven by a simple dilution effect. If group size does affect the cost of inspection, it is therefore likely to do so via a different mechanism such as heterogeneous cost-sharing, or spatial structuring (e.g. [55]; reviewed in [56]). To learn more about the empirical effect of risk perception on risky cooperative behaviour in groups, it will be useful to find ways to directly manipulate risk perception while keeping social factors constant (e.g. using turbidity [57] or conspecific alarm cues [58]). Comparative studies on the effect of group size on participation across cooperative contexts in guppies (e.g. foraging) and in different taxa could highlight patterns that further our understanding of the effects of group size on the importance of risk perception in cooperative decision-making.

Heterogeneity in cost-sharing might be a more biologically relevant extension to the volunteer's dilemma. Heterogeneity among individuals, combined with the high levels of shoaling typical of fish, could help to explain our results because larger groups are more likely to contain individuals whose behaviour is more extreme, for example very bold individuals who inspect more frequently. Shoaling behaviour could then result in the other individuals also inspecting (or at least joining an inspecting party) more frequently as a product of one or a few outlying individuals. High levels of phenotypic variation, which is well documented in guppies, could also help to explain some of the between-group variation that we observed within experimental group sizes in this experiment as interaction among phenotypes has been shown to affect cooperative behaviour [59]. Additionally, while all individuals in the study were laboratory-reared under similar conditions, some of the variation in guppies is likely to be genetically inherited, and thus persist even under shared conditions. Modelling heterogeneity among individuals—for example, to reflect individual differences in personality or experience—has been shown to predict that larger groups should cooperate more frequently in other public goods games (reviewed in [20]). Building on these models, for example by modelling leadership, or other behavioural phenotypes explicitly, could highlight potential mechanisms underpinning a positive group size effect in predator inspection and other natural volunteer's dilemmas.

Another way in which group size might positively influence cooperative behaviour is if the rewards from cooperating increase with group size (i.e. there are ‘synergistic’ benefits; e.g. [60]), which could lead individuals in larger groups to inspect more than those in smaller groups. This could be the case if, for example, the information that individuals gain from predator inspection can be obtained more efficiently in a larger group. There is evidence that animals can combine both social and personal information when making decisions [61], and group size can affect information acquisition, transfer and use, for example speeding up information transmission (e.g. in fish shoals [62]) and reducing the cost of information acquisition (e.g. in sheep flocks [63]). Experimentally investigating how information is acquired and transmitted during predator inspection could identify whether there might be synergistic benefits to individuals inspecting in groups that would explain why individuals in larger groups might be more likely to cooperate in this and other contexts in which the reward is information (or another non-rivalrous resource). High-resolution automated tracking will facilitate these analyses, making novel approaches possible. For example, time series analysis methods and information theoretic approaches (e.g. for identifying leadership (e.g. [64]) or social dominance (e.g. [65])) will allow us to quantify fine-scale animal movement and information transfer during both natural and experimental encounters more precisely than has previously been possible. Models incorporating synergistic benefits into the volunteer's dilemma can predict that individuals in larger groups cooperate more (an example is given in electronic supplementary material, S6.2), suggesting that this effect might be applicable in a broad set of cooperative contexts.

Our finding that experimental group size affected frequency of cooperation but not the duration or distance of inspections suggests that the decision to inspect, and some of the decisions made during inspection, rely on different or changing social cues. Predator inspection has largely been studied from the perspective of decisions made during inspection, when individuals adjust distance and duration seemingly dependent on the behaviour of others (e.g. under the framework of a Prisoner's Dilemma [66,67]). However, the initial decision to inspect—which we consider a volunteer's dilemma—is less well studied (but see [14]). Our contrasting results for frequency of inspection and behaviour during inspection suggest that it will be important in future work to consider the pre-inspection and during-inspection decisions to be part of related but distinct behavioural processes. Modelling predator inspection as a series of decisions will give us a better understanding of the drivers of behaviour at different stages of decision-making. For example, the outcome of the initial volunteer's dilemma (the decision to inspect) might impact the outcome of a subsequent Prisoner's Dilemma (behaviour during inspection). Modelling inspection at a finer scale like this could allow us to make more precise testable predictions about predator inspection behaviour across taxa and develop our understanding of the social processes that can facilitate this and other coordinated and cooperative behaviour in the face of predation risk.

Using group density as a metric, we found that group cohesion was not affected by experimental group size. Further, we found that cohesion did not follow the same patterns, with respect to experimental group size, as time spent in refuges, our other measure of risk perception. We had predicted that risk perception would affect both cohesion and time spent in refuges and, as such, that the two would be affected by experimental group size in the same way. Because cohesion did not change when we introduced the predator stimulus, but time spent in the refuges did, we conclude that group density might not be a good measure of risk perception in this experimental setting. This could have been because individuals in all experimental group sizes formed smaller sub-groups, which were also unaffected by experimental group size or introduction of the predator stimulus. This suggests that the type of social partner might be more important than the number of social partners in the context of predator inspection, and simple density- and distance-based measures might not capture these aspects of social behaviour. Theoretical studies suggest that partner choice can be an important driver of the evolution of cooperative behaviour (e.g. [10,68]), and experiments and observations of guppies show that social ties are important, particularly in the presence of a threat [38,59,69]; preference for familiar individuals can be strong in the group sizes that we tested (≲ 20 individuals) [70]. This could explain why our cohesion measures did not relate to either experimental group size or introduction of the predator stimulus. Other measures of cohesion, such as alignment and speed-matching, might capture aspects of social behaviour that are more relevant for predator inspection and other such highly coordinated collective behaviours.

## 5. Conclusion

Contrary to the prediction of the volunteer's dilemma, individuals in larger groups are not necessarily less cooperative than those in smaller groups—we found that individuals in large experimental groups actually cooperated more frequently in cooperative anti-predator behaviour than those in smaller groups. This suggests that current models of the volunteer's dilemma (and its extensions, e.g. cost-sharing) do not fully capture cooperation during anti-predator behaviour. To better understand predator inspection and other cooperative anti-predator behaviours, it will be valuable to quantify more fine-scale details of behaviour both before and during cooperative events, acknowledging that these behaviours involve many decisions, potentially based on different cues. Investigating information transfer during behaviours such as predator inspection and mobbing will also be useful for understanding the relative effects of social and private information in these behaviours and the impact of social context on these measures. Further extensions of the volunteer's dilemma that incorporate, for example, heterogeneity among group members or social dynamics (e.g. pair bonds) are likely to be applicable to cooperative anti-predator behaviour in a broad range of taxa, and to other public goods scenarios such as cooperative hunting. Such extensions could highlight the conditions required for group size to positively impact cooperation as we have observed. More broadly, our experiment highlights the importance of testing theory explicitly and in a variety of contexts. Using empirical data to inform the development of models that remain simple enough to improve our understanding, but biologically relevant enough to make accurate qualitative predictions about specific systems will allow us to develop a more complete view of cooperative behaviours in a broad variety of systems.

## Data Availability

Data are available from the Dryad Digital Repository: https://doi.org/10.5061/dryad.2fqz612v3 [40]. Additional information is provided in electronic supplementary material [72].
